# Measuring Enzymatic HIV-1 Susceptibility to Two Reverse Transcriptase Inhibitors as a Rapid and Simple Approach to HIV-1 Drug-Resistance Testing

**DOI:** 10.1371/journal.pone.0022019

**Published:** 2011-07-20

**Authors:** Dieter Hoffmann, Albert D. Garcia, P. Richard Harrigan, Ian C. D. Johnston, Tadashi Nakasone, J. Gerardo García-Lerma, Walid Heneine

**Affiliations:** 1 Laboratory Branch, Division of HIV/AIDS Prevention, National Center for HIV, STD, and TB Prevention, Centers for Disease Control and Prevention, Atlanta, Georgia, United States of America; 2 British Columbia Centre for Excellence in HIV/AIDS, Vancouver, British Columbia, Canada; 3 Miltenyi Biotec, Bergisch Gladbach, Germany; 4 AIDS Research Center, National Institute of Infectious Diseases, Tokyo, Japan; University of Cape Town, South Africa

## Abstract

Simple and cost-effective approaches for HIV drug-resistance testing are highly desirable for managing increasingly expanding HIV-1 infected populations who initiate antiretroviral therapy (ART), particularly in resource-limited settings. Non-nucleoside reverse trancriptase inhibitor (NNRTI)-based regimens with an NRTI backbone containing lamivudine (3TC) or emtricitabine (FTC) are preferred first ART regimens. Failure with these drug combinations typically involves the selection of NNRTI- and/or 3TC/FTC- resistant viruses. Therefore, the availability of simple assays to measure both types of drug resistance is critical. We have developed a high throughput screening test for assessing enzymatic resistance of the HIV-1 RT in plasma to 3TC/FTC and NNRTIs. The test uses the sensitive “Amp-RT” assay with a newly-developed real-time PCR format to screen biochemically for drug resistance in single reactions containing either 3TC-triphosphate (3TC-TP) or nevirapine (NVP). Assay cut-offs were defined based on testing a large panel of subtype B and non-subtype B clinical samples with known genotypic profiles. Enzymatic 3TC resistance correlated well with the presence of M184I/V, and reduced NVP susceptibility was strongly associated with the presence of K103N, Y181C/I, Y188L, and G190A/Q. The sensitivity and specificity for detecting resistance were 97.0% and 96.0% in samples with M184V, and 97.4% and 96.2% for samples with NNRTI mutations, respectively. We further demonstrate the utility of an HIV capture method in plasma by using magnetic beads coated with CD44 antibody that eliminates the need for ultracentifugation. Thus our results support the use of this simple approach for distinguishing WT from NNRTI- or 3TC/FTC-resistant viruses in clinical samples. This enzymatic testing is subtype-independent and can assist in the clinical management of diverse populations particularly in resource-limited settings.

## Introduction

Antiretroviral therapy has significantly improved life expectancy and quality of life in persons living with HIV [1]. Currently there are 24 antiretroviral drugs approved by the US Food and Drug Administration (FDA) for the treatment of HIV-1-infected persons, including 11 reverse transcriptase (RT) inhibitors, 10 protease inhibitors, 1 fusion inhibitor, 1 entry inhibitor, and 1 integrase inhibitor. The selection of a combination regimen that maximally suppresses virus replication is critical for treatment success, since persistent virus replication due to suboptimal therapy may result in the selection of viruses carrying drug-resistance mutations. The emergence of drug-resistant viruses can be one of the most important factors leading to therapy failure [2]. Accumulating data from various retrospective and prospective studies support the use of drug-resistance testing in many clinical situations, and several agencies and expert panels such as the IAS-USA Panel [3], the EuroGuidelines Group for HIV Resistance [4], and the U.S. Department of Health and Human Services (http://www.aidsinfo.nih.gov/ContentFiles/AdultandAdolescentGL.pdf) recommend drug-resistance testing for the management of antiretroviral therapy.

NNRTI-based ART regimens containing efavirenz (EFV) or nevirapine (NVP) are frequently used in first regimens worldwide. These regimens typically include a nucleoside RT inhibitor backbone containing either lamivudine (3TC) or the closely related emtricitabine (FTC). Resistance to 3TC/FTC is primarily associated with mutations at position 184 of the HIV-1 RT, in which the wild-type (WT) Methionine (M) is frequently replaced by Valine (V) and less commonly by Isoleucine (I). The presence of the M184V mutation results in >100-fold decreased susceptibility to both drugs [5], [6]. EFV and NVP have overlapping resistance profiles conferred by a number of mutations. K103N and Y188L confer high-level resistance to NVP and EFV, while Y181C/I/V and G190A mainly reduce susceptibility to NVP [7]–[9]. Virologic failure with NNRTI-containing regimens generally associates with the emergence of NNRTI- and/or 3TC/FTC-resistant viruses [10], [11]. In one study of drug-naïve persons comparing EFV with either Combivir (zidovudine/3TC) or Truvada (tenofovir and FTC), treatment failures at 96 weeks had viruses that were more commonly NNRTI-resistant or 3TC/FTC-resistant than tenofovir- resistant [12]. Likewise Margot et al. found K103N as the most common resistance mutation in patients failing regimens containing tenofovir, FTC and efavirenz or zidovudine, 3TC and EFV [13]. M184V and K103N/Y181C were seen in >10% of patients failing antiretroviral therapy in British Columbia, Canada during 1996 to 2003 [14]. Delaugerre et al. detected NNRTI-associated mutations in more than 98% of patients failing an efavirenz- or NVP-containing regimen [8].

Therefore, the availability of simple assays to measure NNRTI or 3TC/FTC resistance can be highly useful for managing first-line regimens. Rapid assays that can distinguish WT from 3TC/FTC- or NNRTI- resistant virus during virologic failure can inform decisions for switching regimens, which is particularly important in resource-limited settings with often single second-line regimens. Although sequencing is a widely used genotypic test to monitor drug resistance in resource-rich countries, the complexity and cost of this testing limits its utility for resource-limited countries with large HIV-infected populations. Thus, current treatment guidelines in resource-limited countries do not include resistance testing. We have previously described the use of a sensitive biochemical assay (Amp-RT) to measure the enzymatic activity of reverse transcriptase (RT) of HIV-1 in plasma and assess its susceptibility to antiretroviral drugs [15]. Like other RT assays that are broadly reactive on all retroviruses, Amp-RT can detect generically RT activity from diverse retrovirus groups including HIV-1 non-subtype B and HIV-2 [15], [16]. We have previously demonstrated that this technology can also be used for HIV drug-resistance testing and have shown that biochemical detection of NNRTI and 3TC resistance by this method correlates well with genotypic and replication-based phenotypic assays [9], [17]. Here, we describe the development of improved Amp-RT assays that use real-time PCR detection and simplified virus capture from plasma. The real-time PCR readout replaces the previously used ELISA-based detection of PCR products and provides a rapid and cost-effective quantitation. Likewise we demonstrate the utility of virus capture from plasma by magnetic beads that eliminate the need for ultracentrifugation. We evaluated Amp-RT-based assays that screen for resistance to 3TC and NVP in a large collection of clinical specimens with known drug-resistance profiles, and show data that support the promise of this testing strategy for drug-resistance management.

## Materials and Methods

### Ethics Statement

Specimens were anonymous residual diagnostic material from subjects who provided written consent for HIV testing. The CDC Institutional Review Board determined that this testing did not involve identifiable human subjects and has approved the study.

### Assay Optimization

The optimization of assay conditions was done by unblinded testing of 140 plasma samples. They consisted of 114 subtype B and 26 non-B subtypes (two A, 22 recombinant AG, two unknown). In addition, the specificity was evaluated by testing 160 samples from HIV- seronegative blood donors and 14 HIV-seropositive specimens that had undetectable RNA viral loads (<40 copies/ml). Assay validation was done by blinded testing 253 (229 subtype B, 14 subtype C, 6 subtype A, 1 subtype D, and 3 recombinant including CRF nomenclatures AG and AE) plasma samples collected at the BC Centre for Excellence in HIV/AIDS (BC, Canada). The median RNA virus load in these samples was 4.28+/−0.84 log_10_ copies/ml and ranged between 2.29 and 5.88.

### Viral load Measurement and Sequencing

Roche Amplicor Version 1.5, with a limit of detection of 50 copies/ml was used for quantitating HIV RNA in plasma. Standard population-based sequencing was performed with a limit of detection of approximately 20–30% for a minority sequence variant as described in [18]. The region sequenced typically covered codon 1 to 400 of the RT.

### Reference Viruses and Drugs

The following isolates were used as control reference viruses in each experiment: HXB2, xxBRU_pitt_, and HIV-1_SUM 9_ as WT viruses; M184V_pitt_ as 3TC-resistant virus carrying the M184V mutation [13]. X403-4 and X82-5 are NVP-resistant isolates carrying the Y181C or K103N/Y181C mutations, respectively [9]. Nevirapine was obtained from the NIH AIDS Research & Reference Program (Rockville, MD). 3TC-triphosphate was synthesized by Sierra Bioresearch (Tucson, AZ). Both drugs were aliquoted and kept at −80°C.

### Principle of the Amp-RT Assay

Amp-RT is an ultrasensitive RT assay that uses PCR to detect RT-generated DNA of an exogenous non-retroviral heteropolymeric RNA template from the encephalomyocarditis virus (EMCV) genome [15]. Levels of EMCV DNA generated by HIV-1 RT in a plasma specimen are quantified by real-time PCR as described below.

### RT Activity Detection by Amp-RT

To detect RT activity in plasma specimens, 250 µl of plasma was first clarified at 10,000 g for 5 min and then ultracentrifuged at 99,000 g for 1 h at 4°C. Virus pellets were concentrated in a final volume of 100 µl of 1× RTCDR buffer (50 mM Tris HCl, 50 mM KCl, 10 mM MgCl_2_, 0.5 mM EGTA, 2 mM DTT, 0.06% NP-40). A known amount of HXB2 particles (1×10^5^) spiked in an HIV-1 seronegative plasma was included in each experiment to control for the quality of the ultracentrifugation procedure. The mean and standard deviation were determined in 35 runs. The range for the control was defined as mean ±3× standard deviation. Thus an individual ultracentrifugation run was considered valid when the control with 1×10^5^ HXB2 particles yielded a Ct value between 18.37 and 28.97.

To eliminate the need for ultracentrifugation, an alternative method was evaluated for virus capture from plasma by using magnetic beads coated with CD44 monoclonal antibody (µMultiMACS™ VitalVirus HIV Isolation Kit; Miltenyi Biotec, Auburn, CA). CD44 is one of the host cell proteins present on HIV l particles [19]. CD44 been shown to be suitable for capture of HIV-1 particless from patient samples [20] and can be used for virus genotyping even from patients with no detectable viral load [21]. After clarifying the plasma at 10,000 g for 5 min, 200 µl were incubated with 20 µl microbeads for 10 min at room temperature. Then, the mixture was applied to MACS separation columns and washed 4 times. HIV-1 particles bound to the column were dissolved in 100 µl 1×RTCDR, yielding HIV RT in enzymatically active form. The eluate was immediately used in the RT reaction or stored at −70 °C.

Levels of RT activity were measured in duplicate using 96-well plates. Briefly, 4 µl of virus pellets or eluants were added to 16 µl of RT buffer containing 50 mM Tris HCl, 50 mM KCl, 10 mM MgCl_2_, 0.5 mM EGTA, 2 mM DTT, 10 µM dCTP, 120 µM dATP, dGTP, and dTTP, 1 µM antisense primer 262R (5′-CAACGTCTTCAAGCGTCGAAT-3′), 0.20 ng × µl^−1^ EMCV RNA, 0.10 u × µl^−1^ RNasin, and 0.06% NP-40. As a competitive inhibitor, 3TC-TP inhibits RT activity more efficiently when dCTP concentration is reduced resulting in less 3TC-TP in the assay. We have previously shown by titrations of dCTP and 3TC-TP that 10 µM of dCTP was optimal for detecting both RT activity and its inhibition by 3TC-TP [17]. The RT reaction was carried out for 2 h at 37°C, followed by a 5 min inactivation of HIV-1 RT at 95°C. To evaluate the susceptibility of the RT for 3TC and NVP, parallel RT reactions were done in the presence of 50 µM 3TC-triphosphate (3TC-TP) and 50 µM NVP.

### cDNA Quantification by Real-Time PCR

Levels of RT-generated EMCV cDNA were quantified by real-time PCR in the TaqMan™ format. Briefly, 10 µl of RT-generated EMCV cDNA were added to 40 µl of PCR buffer containing 1× AmpliTaq Gold buffer, 4.0 mM MgCl2, 250 µM dNTP, 0.5 µM sense primer EMCF1 (5′-CTCCCATCAGGTTGTGCAGCGACC-3′), 0.5 µM antisense primer 262R, 0.192 µM FAM-labeled probe 158T (5′-AAGGTGAGAGCAAGCCTCGCAAAGACA-3′), and 0.05 U × µl^−1^ AmpliTaq Gold polymerase. The PCR reaction mixtures were then subjected to 45 cycles at 95°C for 15 s and 60°C for 1 min. Oligonucleotides EMCF1 and 262R, as well as the probe 158T were obtained from Scientific Resources Program, CDC.

### Measuring Enzymatic RT Resistance to Lamivudine and Nevirapine

Susceptibility of HIV-1 RT for 3TC-TP or NVP was measured by comparing the level of inhibition of the Amp-RT signal in reactions done in the presence of drug relative to reactions without drug. Samples were categorized as sensitive or resistant based on the differences in Ct values (ΔCt) seen in reactions done in the presence and absence of drug. One screening concentration was established for 3TC and NVP based on the results from the 140 samples tested unblinded. The screening concentration was selected to best differentiate in “sensitive” and “resistant” corresponding to the respective genotype. We considered only samples that had an average Ct_0_ value below 36.25 cycles to prevent misinterpretation of resistant samples because of signal instability close to the detection limit. Samples with enzymatic susceptibility data that did not correlate with resistance mutations were tested further to determine Amp-RT IC50 and IC90 values. To this end we applied inhibitor concentrations ranging from 5 to 500 µM, depending on the ΔCt obtained with 50 µM inhibitor concentration. IC50 values were determined by linear regression.

### Statistics

Statistical evaluation and graphic representation of the data was done with with GraphPad Prism 4 (GraphPad Software Inc., USA). We derived ΔCt cut-off values for differentiating between sensitive and resistant from the 140 samples tested unblinded for validation. For 3TC and NVP we defined the cut off value as the mean ΔCt of all genotypically resistant specimens using the screening concentrations for 3TC and NVP plus 3× the standard deviation.

## Results

### Correlation between RT Levels by Amp-RT and RNA Virus Loads

We first tested 140 clinical isolates with known RT genotypes. The median log_10_ RNA virus loads of the 114 subtype B samples was 4.2±0.8 log_10_ (range 2.4–5.5) and correlated well with the Ct values determined by Amp-RT (r = 0.68, 95% confidence interval, 0.77–0.57, *P*<0.0001). A similar correlation between the median log_10_ RNA virus loads and Ct values measured Amp-RT was seen in the 26 non-B subtypes samples (r = 0.69, 95% CI interval, 0.85–0.41, *P*<0.0001), illustrating that Ct values measured by Amp-RT represent a good indication of plasma virus loads. (data not shown). The specificity of the assay was evaluated by testing 160 samples from HIV-seronegative blood donors. The results showed a mean Ct_0_ value of 44.6±1.1 (range 40.2, >45). A similar mean Ct_0_ value (43.8±1.9, range 39.6−45) was seen in 15 HIV-seropositive specimens that had undetectable RNA virus loads (<50 Geq × ml^−1^). Sixty control reactions done in the absence of RT enzyme yielded no signal within 45 PCR cycles. Thus, no RNA-negative sample yielded lower Ct values than the threshold for clinical samples to be included in the resistance evaluation (36.25).

### Detection of 3TC and Nevirapine Resistance

**Figure 1:**
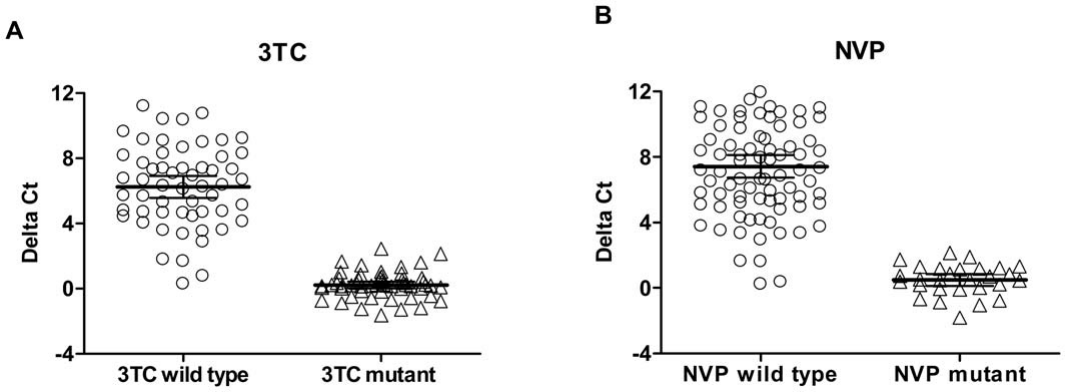
RT inhibition in 140 clinical isolates after virus concentration by ultracentrifugation. Genotypes had been classified into wild type and mutant. Scatter plots show delta Ct values (threshold cycle difference between inhibited and uninhibited reaction) measured with 50 µM 3TC (A) and NVP (B). Horizontal bars indicate the respective mean values and the 95% confidence intervals. For mutants delta22Ct values are close to 0 for both drugs illustrating that mutant RT enzymes are virtually not inhibited by 50 µM drug concentrations.

We next evaluated the ability of the assay to discriminate between 3TC- and NVP-sensitive and resistant viruses. Figure 1 shows the differences of Ct values (ΔCt) for 3TC and NVP seen in all the isolates tested unblinded. The mean ΔCt for 3TC in 56 WT isolates was 6.2±2.5 (range 0.29–11.2) and was significantly higher (*P*<0.0001) than the mean ΔCt seen in the 55 3TC-resistant isolates that had the M184V mutation (0.2±0.8, range -1.6–2.1, Figure 1A). Figure 1B shows that the mean ΔCt for NVP seen in 83 WT isolates was 7.42±3.18 (range 0.235–15.9) compared to a mean ΔCt value of 0.484±0.963 (range -1.85–2.15) seen in 28 NVP-resistant samples carrying K103N, Y181C/I, Y188L, or G190A/Q (*P*<0.0001). These findings confirm that a single drug concentration of 50 µM 3TC or NVP can reliably differentiate between WT and resistant isolates as a screening assay.

We next selected the respective ΔCt cut-off values based on the mean ΔCt+3.0 SD observed in all the mutant isolates, resulting at 2.66 and 3.28 for 3TC and NVP. Using these ΔCt cut-off values, we found a very high concordance between Amp-RT phenotype and RT genotype. For instance, all 55 samples that had M184V were classified as 3TC-resistant while all 28 samples carrying NVP-associated mutations were considered NVP-resistant. On the other hand, 52 of the 56 samples with 184 M (92.9%) were interpreted 3TC-sensitive, while 79 of the 83 samples lacking detectable NVP-associated mutations (95.2%) were classified as NVP-sensitive. Of the 8 discordant samples (4 for 3TC and 4 for NVP), five had ΔCt values close to the assay cut-offs (data not shown).

### Assay Variability

We evaluated inter-assay variability of ΔCt values by comparing the values observed in our control WT, 3TC- and NVP-resistant isolates tested in 14 independent experiments. The ΔCt values for HXB2 WT ranged from 7.9 to 10.8 with a mean of 9.5 for 3TC, and from 7.9 to 11.9 for NVP (mean 9.73). The 3TC-resistant control strain M184V_pitt_ yielded a mean ΔCt of 0.9 (range 0.3–1.6) for 3TC and 8.8 for NVP (range 6.9–11.0). On the other hand the NVP-resistant strain Y188C X403-4 resulted in a mean of 8.4 (range 6.9–9.7) for 3TC and 1.9 (range 1.3–2.7) for NVP. The variability was much lower than the ΔCt cut-off values for the screening assays. Thus no sample will be erroneously classified sensitive because of assay variation.

### Assay Performance on Clinical Samples

We analyzed 173 specimens blinded, with genotypes revealed only after the testing. Applying our previously defined cut-off values, 99 samples were classified as 3TC-sensitive and 74 as 3TC-resistant (Table 1) by Amp-RT. Of the 99 samples 3TC-sensitive by Amp-RT, 94 (96%) were genotypically WT, 3 had a mixture of M184 M/V, and 2 showed M184 V. The two samples with M184 V had ΔCt values of 2.7 and 2.8, which were close to the assay cut-off (2.66). Of the 8 samples that had an M184 M/V mixture, 5 were Amp-RT resistant and 3 had ΔCt values below 4.1. Of the 74 samples with phenotypic resistance to 3TC, 70 (94%) had the M184 V or M184I mutations. Among these samples, there were 64 184 V, 1 had 184I, and 5 had 184 V/M mixtures. In four samples, phenotypic resistance to 3TC was seen in the absence of detectable 184 V or 184I. We tested two of them (ΔCt values 0.8 and 1.2) with higher 3TC concentrations (150, 500, and 1000 µM). The results further confirmed reduced 3TC susceptibility in these two isolates (data not shown). However, the levels of 3TC resistance were lower than those seen in an isolate carrying the 184 V mutation. In three of the four strains with reduced enzymatic susceptibility to 3TC in the absence of 184I/V we found the mutation V118I which has been previously associated with low-level enzymatic resistance to 3TC-TP [22].

**Table 1:** Blinded drug-resistance testing with the 50 µM screening format after virus enrichment by ultracentrifugation.

	3TC			NVP		
Genotype	Wild type	Mixed genotype	Mutant^a^	Wild type	Mixed genotype	Mutant^b^
Amp-RT sensitive	94	3	2	73	7	2
Amp-RT resistant	4	5	65	3	11	77
Total	98	8	67	76	18	79

a including the mutations M184V/I.

b including the mutations K103N, Y181C/I, Y188L, G190A/Q, or K238N.

Of the 173 samples tested for NVP resistance, 82 were classified as sensitive and 91 as resistant. Enzymatic NVP resistance was associated with the presence of K103N, Y181C/I, Y188L, G190A/Q, or K238N in 88 of the 91 samples. The other 3 samples showed NVP resistance in the absence of any of these 5 mutations. Seventy-three of the 82 samples classified as NVP sensitive had WT genotypes. Of the other remaining 9 samples, 2 had the K103N mutation and a ΔCt value close to the assay cut-off (3.8 and 3.4), and 7 had mixtures of WT and mutant genotypes. Overall, the current findings demonstrate the ability of the new Amp-RT format to detect phenotypic resistance to 3TC or NVP.

### Assay Performance on Bead-Captured HIV-1

**Figure 2:**
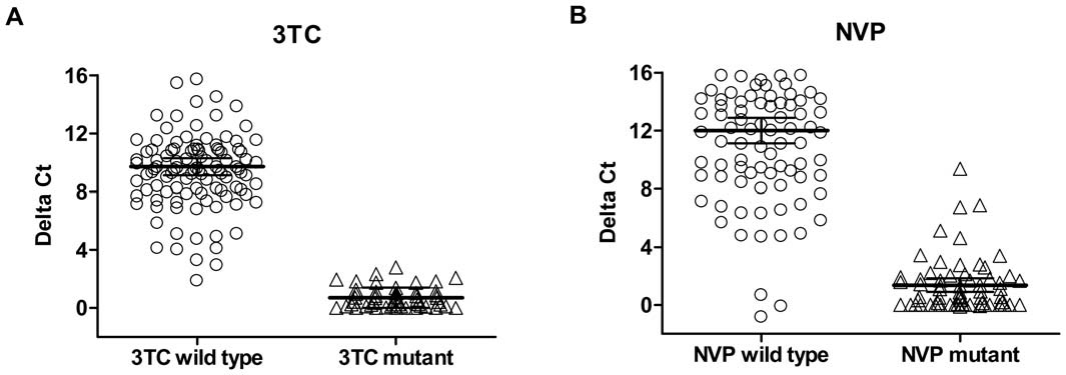
RT inhibition in 162 samples after virus concentration with CD44-coated beads. Genotypes had been classified into wild type and mutant. Scatter plots show delta Ct values (threshold cycle difference between inhibited and uninhibited reaction) measured with 50 µM 3TC (A) and NVP (B). Horizontal bars indicate the respective mean values and the 95% confidence intervals. The established cut-offs for both drugs classified the isolates as summarized in Table 2. Data correlate well to those obtained by ultracentrifugation (Figure 1) with slightly more discrepancies between geno- and phenotype for NVP.

We also tested 162 blinded samples with a simplified protocol replacing ultracentrifugation by concentrating the virus with CD44-coated magnetic beads (µMultiMACS™ VitalVirus HIV Isolation Kit). The summarized results are illustrated in scatter plots in Figure 2. Our cut-off values categorized the samples as shown in Table 2. Of 110 samples categorized sensitive for 3TC by Amp-RT only one had the mutation M184V, the corresponding ΔC of 2.79 was only slightly above the cut-off (Figure 2A). Conversely, 52 were enzymatically resistant to 3TC with 51 strains harboring M184V. For NVP 102 specimens were Amp-RT sensitive (Figure 2B). No NVP resistance mutation had been found in 95, one had a mixed genotype K103 K/N and 6 contained the mutations K103N, G190A, or Y188L. Four of the six samples were further analyzed with different NVP concentrations to allow for IC50 determination. Fold changes in IC50 values relative to WT HIV-1_HXB2_ were 9.8, 6.4, 3.4, and 1.0 indicating enzymatic NVP resistance for three 3 of 4 specimens. Fifty-nine specimens were found to be enzymatically NVP-resistant. Of these, 56 had NNRTI mutations and 3 had no NNRTI-resistance mutations. In summary, there was a good correlation between genotype and enzymatic resistance in both formats of virus preparation.

**Table 2:** Blinded drug-resistance testing with the 50 µM screening format after virus enrichment by CD44-coated beads.

	3TC			NVP		
Genotype	Wild type	Mixed genotype	Mutant^a^	Wild type	Mixed genotype	Mutant^b^
Amp-RT sensitive	109	0	1	95	1	6
Amp-RT resistant	1	0	51	3	0	56
Total	110	0	52	98	1	62

a including the mutations M184V/I.

b including the mutations K103N, Y181C/I, Y188L, G190A/Q, or K238N.

## Discussion

Current treatment guidelines in the developed world recommend the use of drug-resistance testing for the management of patients receiving antiretroviral therapy. However, because such testing relies mainly on sequencing or recombinant virus phenotyping, which are costly and complex, it has remained largely unavailable for the vast majority of patient populations receiving ART in resource-limited settings. Thus, simpler and less expensive drug resistance technologies may improve patient management. Previously explored technology relied on detecting and phenotyping HIV-associated RT activity in plasma by ultrasensitive biochemical assays [9], [17], [23], [24]. In this study we describe an improved biochemical approach to resistance testing for NNRTI and 3TC/FTC. The principle is based on our previously reported Amp-RT assays [9], [17] but has been improved in two major ways. First, we include real-time PCR for the detection of Amp-RT product which eliminates the need of ELISA quantitation of the PCR products, thus reducing time and cost. Secondly, we describe a method of HIV capture from plasma by magnetic beads, eliminating the need for ultracentrifugation and reducing cost and complexity.

Conventionally, phenotypic resistance is reported as fold changes in IC50 values over a reference WT virus. Since this approach requires testing of multiple drug concentrations we sought to evaluate a screening method with only one drug concentration to help simplify testing. We have found previously that this approach was feasible, given the high level of resistance conferred by M184V and many NNRTI mutations. By testing large numbers of mutant and WT clinical specimens we show here that inhibition with the 50 µM screening concentration correlated well with the absence of genotypic markers of resistance to 3TC/FTC. This screening concentration is close to the 3TC-TP concentration that inhibits WT enzymatic activity by 100% (IC100). We demonstrate that enzymatic 3TC resistance was strongly associated with M184I/V. In addition, we also found that the mutation V118I was associated with enzymatic 3TC resistance in the absence of M184V in 3 of 4 cases. In vitro, V118 is reported to confer resistance to 3TC and other nucleoside analogues only in combination with other mutations like D67N and T215Y [22]. However, consistent with our results, Girouard and colleagues detected a 21.7-fold enzymatic resistance to 3TC-TP in RTs harboring just V118I [25]. The clinical significance of this resistance is unclear and requires more study. NNRTI mutations are common in ART-treated populations. Our data also show that the screening Amp-RT format detecting resistance to NVP correlated with the presence of K103N, Y181C/I, Y188L, and G190A/Q. Thus, all primary mutations conferring resistance to NNRTIs are detected. While K103N or Y188L confer high cross-resistance between NVP and EFV[7], Y181C/I/V is associated with only moderate resistance to EVF. Thus, the use of NVP in the assay allowed the detection of resistance mutations that may be selected by either NVP or EFV. However, further work may be needed to determine if EFV is more suitable than NVP for detecting resistance by particular NNRTI mutations that confer higher resistance to EFV than NVP and whether the use of EFV would have detected resistance in few mutant samples that showed WT results in the current assay.

Despite the strong correlation between genotype and enzymatic resistance, few specimens showed discrepant results. The reasons for the discrepancies are not clear but could be related to a number of factors including a wrong genotype because of sequencing error, imperfect cut-offs values, mutations currently not known to cause enzymatic resistance, or mixed genotypes that are inconsistently detected genotypically or enzymatically. While enzymatic IC50 determination clarified the discrepancies in many samples, further phenotypic testing by culture-based methods and additional genotypic analysis by ultradeep sequencing for minority or mixed resistance, and inclusion of mutations in the connection domain in RT that confer NNRI resistance could help resolve the source of the residual discrepant results [26].

Although this work has focused on assays for 3TC/FTC and NNRTI resistance because of the high prevalence in virologic failure and the high level of resistance, this testing approach can be adapted to other RT inhibitors, including new-generation NNRTIs and other nucleoside analogs. Such assays will require careful development that takes into account the level of enzymatic resistance found in the mutants, and the need for a screening or IC50-based testing format. One important improvement in our testing is the capture of virus by magnetic beads, which eliminated the need for ultracentrifugation and the associated cost, labor, and maintenance. We demonstrate the utility of virus capture with magnetic beads containing antibodies against CD44, a host-cell protein that is commonly incorporated on the surface of HIV-1 particles. This method has the advantages of allowing high throughput and automated processing. Overall, in contrast to conventional sequencing or cell-culture based phenotypic assays, the enzymatic Amp-RT testing described in this work does not require special and expensive instrumentation or equipment but instead a real-time PCR cycler and common equipment available in clinical virology laboratories with biosafety level 2 capacity. Additional studies geared to evaluating the assay in a resource-limited setting would include further simplified storage conditions, sensitivity to repeated freezing and thawing of the reagents and interference with other infectious agents present in the samples. The described assays have a short turnaround time and have the advantage of being subtype-independent. The reaction carried out in the absence of drug can also be used as a marker of virus load as demonstrated in the good correlation with RNA loads in this and previous studies [23]. In conclusion, we demonstrate the strong potential for a simplified drug-resistance testing strategy based on sensitive enzymatic assays for two key drug-resistance profiles that are of particular importance for first-line regimens. Our data strongly support expanded clinical evaluation and standardization of this testing approach.
